# Effects of Nitrogen and Phosphorus Inputs on Soil Bacterial Abundance, Diversity, and Community Composition in Chinese Fir Plantations

**DOI:** 10.3389/fmicb.2018.01543

**Published:** 2018-07-19

**Authors:** Qing Wang, Cong Wang, WeiWei Yu, Ali Turak, Diwen Chen, Ying Huang, Junhua Ao, Yong Jiang, Zhengrui Huang

**Affiliations:** ^1^Guangdong Provincial Bioengineering Institute (Guangzhou Sugarcane Industry Research Institute), Guangdong Key Laboratory of Sugarcane Improvement and Biorefinery, Guangzhou, China; ^2^State Key Laboratory of Urban and Regional Ecology, Research Center for Eco-Environmental Sciences, Chinese Academy of Sciences, Beijing, China; ^3^Guangdong Key Laboratory of Integrated Agro-environmental Pollution Control and Management, Guangdong Institute of Eco-environmental Science and Technology, Guangzhou, China; ^4^Science and Technology Management Department, Sinofert Holdings Ltd., Chemsunny World Trade Center, Beijing, China

**Keywords:** soil bacterial community, 16S rRNA gene, N and P inputs, Illumina Miseq sequencing, Chinese fir plantation

## Abstract

Nutrient inputs to forest ecosystems significantly influence aboveground plant community structure and ecosystem functioning. However, our knowledge of the influence of nitrogen (N) and/or phosphorus (P) inputs on belowground microbial communities in subtropical forests is still unclear. In this study, we used quantitative polymerase chain reaction and Illumina Miseq sequencing of the bacterial 16S rRNA gene to investigate bacterial abundance, diversity, and community composition in a Chinese fir plantation. The fertilization regimes were as follows: untreated control (CK), P amendment (P), N amendment (N), and N with P amendment (NP). Additions of N decreased soil pH and bacterial 16S rRNA gene abundance by 3.95 (from 4.69 to 3.95) and 3.95 × 10^9^ copies g^−1^ dry soil (from 9.27 × 10^9^ to 3.95 × 10^9^ g^−1^ dry soil), respectively. Bacterial richness and diversity decreased with N addition (N and NP) rather than only P input. *Proteobacteria*, *Acidobacteria*, and *Actinobacteria* were the major phylum across all treatments. Nitrogen addition increased the relative abundance of *Proteobacteria* and *Actinobacteria* by 42.0 and 10.5%, respectively, while it reduced that of *Acidobacteria* by 26.5%. Bacterial community structure in the CK and P treatments was different from that in the N and NP treatments upon principle coordinates analysis. Phosphorus addition did not significantly affect soil bacterial communities, and no interactions between N and P inputs on microbial traits were observed. Soil pH and mineral N availability appeared to have a cooperative effect on bacterial abundance and community structure, with soil pH being the key influencing factor by canonical correspondence analysis. These results indicate that inorganic N rather than P fertilization affected both bacterial abundance and community composition in subtropical forests.

## Introduction

Soil nutrient inputs such as nitrogen (N) and phosphorus (P) are critical to control plant growth in tropical forest plantations (Chen et al., 2015). Because of high amounts of fossil fuels burning and overuse of chemical fertilizers in agricultural production, N deposition has been increasing significantly in the past 100 years (IPCC, 2013), especially in the warm and humid climatic zone in Asia (Dentener et al., 2006). Nitrogen is considered as a limiting factor that influences the plant biodiversity and primary productivity in forest ecosystems (Bobbink et al., 2010; Weand et al., 2010). In addition, chronic elevated N input has been shown to lead to many adverse impacts, including soil acidification (Guo et al., 2010; Mao et al., 2017), nutrients imbalance (Lu et al., 2010), and increased greenhouse gas emissions (IPCC, 2007). The soils in Chinese subtropical forests are highly weathered because of high temperature and precipitation, resulting in a large amount of available P being lost (Li et al., 2014; Cui et al., 2017). Thus, P is always scarce in subtropical forests (Zhao et al., 2014; Tang et al., 2016). Moreover, N input may result in element imbalance and further aggravate P limitation in the forest ecosystem (Tang et al., 2016). The continuous anthropogenic P additions are essential to improve forest primary production and little decomposition in forest ecosystems (Cleveland et al., 2006; Kaspari et al., 2008). Nitrogen and phosphorus additions may impact the abundance, diversity, and community composition of soil microbes, which play an important role in regulating soil fertility (van der Heijden et al., 2008). However, effects of repeated N and P additions on belowground ecosystems such as soil bacterial communities in subtropical forest ecosystems remain poorly understood.

Soil microorganisms are important drivers of energy flow and nutrient cycling, such as carbon (C), N, and P cycling in terrestrial ecosystems (Artursson et al., 2006; Morris and Blackwood, 2015). Therefore, soil functioning always depends primarily on microbial community structure, activity, and stability (Dunbar et al., 2002; Coleman and Whitman, 2005). Soil microorganisms are sensitive to various changes in soil nutrients and pH (Mele and Crowley, 2008; Zhong et al., 2010). It is well known that soil bacterial communities can be a potential ecological indicator of soil quality (Bending et al., 2004). Studying the feedback of soil microorganisms to added N and/or P is very important to understand the effects of global changes on ecosystem processes regulated by soil biota. The influence of N inputs on soil bacterial community structure have been well studied; however, the results of these studies have been inconsistent (Cui et al., 2017; Nie et al., 2018). For example, Freedman et al. (2015) reported that neither the total nor active bacterial community was influenced by N enrichment. However, Nie et al. (2018) found that high N application strongly shaped bacterial community structure and that ammonium availability, rather than pH or nitrate concentration, was a key environmental parameter determining this shaping. These inconsistent results indicate that the responses of soil microbial communities to N addition are highly variable in different forest ecosystems. However, only few studies were conducted to study the response of soil bacterial communities to P amendment in subtropical forest ecosystems. Furthermore, in the context of the increased nitrogen deposition, we also need to understand the influence of P fertilization on soil microbial communities in forest ecosystem that receive exogenous N inputs.

The Chinese fir (*Cunninghamia Lanceolata*), which covers over 9 million ha in China, is the most common coniferous timber species that has been extensively planted in southern China (Tang et al., 2016). In order to improve soil quality, conservation, and enhanced the productivity of Chinese fir plantations, a large number of measures have been applied, among which fertilization is the most effective and feasible (Zhang et al., 2004). A number of field studies have focused on the impacts of nutrients on soil C and N sequestration in Chinese fir plantations (Wei et al., 2012; Fan et al., 2014), but few studies have investigated the belowground soil microbial properties.

To better understand how N, P, and NP additions influence bacterial abundance, diversity, and composition in subtropical forests, we set up a N and/or P amendment trial in a Chinese fir plantation. The specific objectives of this study were to (1) assess the responses of bacterial abundance, diversity, and community composition to N and/or P addition; (2) identify which soil properties were correlated with bacterial community. We hypothesized that (1) the changes in soil pH and mineral N availability induced by N addition might affect bacterial community abundance, diversity, and composition and (2) the increased P content after P addition might not affect these variables of soil bacterial communities.

## Materials and Methods

### Experimental Site, Plots, and Design

This study was conducted in a rehabilitated secondary forest located at the Qianyanzhou Experimental Station, Chinese Academy of Sciences, Taihe County, Jiangxi province, southern China (26°44′52″N, 115°04′13″E). The soil in the forest, which is classified as Typical Hapludult Ultisols (locally “red soil”), developed from Quaternary Red Clay and covers over 60% of the 1.14 million km^2^ of total land area in southern China. The site has a monsoon season and humid climate. The duration of the growing season is about 270 days, and the average annual precipitation and temperature are 1471.2 mm and 17.9°C, respectively (Dong et al., 2015). The annual clear-sky duration and solar radiation are 1306 h and 4349 MJ/m^2^, respectively.

In 2011, 16 experimental plots (20 m × 20 m each) were placed in a random manner with 10-m-wide buffer strip between plots. Four fertilization treatments (four replicates each) were set up in this fir plantation: without fertilizer (CK), P addition (P) (50 kg P ha^−1^ year^−1^), N addition (N) (100 kg N ha^−1^ year^−1^), and N and P addition (NP) (100 kg N ha^−1^ year^−1^ + 50 kg P ha^−1^ year^−1^). Nitrogen and phosphorus fertilizer were applied as NH_4_NO_3_ and NaH_2_PO_4_, respectively. Nitrogen and and/or phosphorus fertilizer were dissolved in 20 L of distilled water and, then, added into the corresponding plots. Twenty liters of distilled water was added into the control plots.

### Soil Sampling and Analysis

Soil cores were collected in late August of 2017. From each replicate plot, 10 subsamples (0–20 cm) were collected using corers, after which they were pooled together to minimize within-plot variation. Subsequently, soil samples were mixed thoroughly and then passed through a 2-mm sieve and separated into two portions. One portion was used for soil chemical analysis and another that was frozen at −80°C for subsequent molecular analysis. The soil pH (water:soil, 5:1) was determined using a pH meter. Soil KCl-extractable NH_4_^+^-N and NO_3_^−^-N were extracted using a ratio of 1:5 (fresh soil: 2 M KCl, w/v) by shaking at 200 rpm for 1 h, and quantified by a Segmented Flow Analyzer (SAN^++^, Skalar, Holland). Dissolved organic carbon (DOC) was extracted by 0.5 M K_2_SO_4_ and determined using a total organic carbon analyzer (Multi N/C 3000; Analytik, Jena, Germany; Watkins et al., 1987). Total nitrogen (TN) was determined using Elemental Analyzer (Elemental Analyzer, Germany). Available phosphorus was extracted using sodium bicarbonate and then measured by the molybdenum-blue method.

### Soil DNA Extraction and quantitative PCR (qPCR) Analysis of Bacterial Abundance

The total DNA was isolated from 0.4 g of soil using MoBio Powersoil^TM^ DNA Isolation Kit (Carlsbad, CA, United States). DNA concentration was checked using a NanoDrop ND-1000 spectrophotometry (United States), then DNA samples were stored at −80°C refrigerator for further analysis.

To estimate the bacterial abundance, quantitative polymerase chain reaction (qPCR) assays were performed using universal eubacterial 16S rRNA gene primers. The forward primer was 338F (5′-ACTCCTACGGGAGGCAGCA-3′), and the reverse primer was 806R (5′-GGACTACHVGGGTWTCTAAT-3′; Fierer et al., 2005). The abundance of bacterial 16S rRNA gene was determined on an ABI7500 (ABI, United States). The 25 μL reaction mixture included 12.5 μL of SYBR Premix Ex Taq^TM^ (TakaRa Biotechnology, Dailian, China), 0.5 μL of each primer (10 mM), 1–10 ng of template DNA. The PCR condition was as follows: 3 min at 95°C, 35 cycles of 40 s at 95°C, 30 s at 54°C, 40 s at 72°C, and a plate read at 83°C for 10 s. The qPCR assay was performed in triplicate for each replicate. The amplification efficiency for all samples ranged between 91 and 100%.

### Bacterial 16S rRNA Gene Illumina Miseq Sequencing and Bioinformatics Analysis

The V3-V4 region of the bacterial 16S rRNA gene was amplified from DNA extracts using the same primers for qPCR. The adapter linked forward primer included a 5-bp barcode was used for sample identification. Each PCR mixture (25 μL) included 0.5 μL of each primer, 2 μL template DNA (1–10 ng), 12.5 μL of Premix Taq^TM^ (2×) (Takara), and 9.5 μL of ddH_2_O. The thermocycling conditions consisted of initial denaturation at 94°C for 5 min, followed by 30 cycles of denaturation at 94°C for 40 s, annealing at 54°C for 30 s, and elongation at 72°C for 6 min. PCR products were purified with a QIA quick Gel Extraction kit (QIAGEN, Germany). The high-throughput sequencing was conducted on the Illumina MiSeq 2500 platform (Illumina, San Diego, CA, United States).

Sequence data including raw data and clean data were merged and filtered using Mothur (Schloss et al., 2009). Briefly, the raw sequences were sorted and distinguished by unique 5-bp barcodes, and those shorter than 300 bp were removed. The barcodes and primer sequences were then trimmed (Supplementary Table S2), after which the remaining high-quality sequences with ≥97% similarity were clustered into operational taxonomic units (OTUs; Edgar, 2013). Representative sequences for each OTU were then assigned to taxonomy by the Ribosomal Database Project (RDP) classifier (Wang et al., 2007). All the sequence data were deposited into the NCBI Sequence Read Archive database under accession numbers SRR6263277-SRR6263292. Observed OTU numbers, Pielou’s evenness, Chao1 richness, and ACE evenness, as well as Shannon’s diversity indices, were calculated using the Mothur software to estimate bacterial alpha diversity. Beta diversity was calculated based on Bray-Curtis distance matrices.

### Statistical Analysis

One-way ANOVA was conducted to compare the effects of different fertilization treatments on bacterial 16S rRNA gene abundance and alpha diversity indices using SPSS version 19.0 (SPSS, Chicago, IL, United States). The mean values of four replicate plots were compared using Duncan’s test when a significant *F*-value was obtained (*p* < 0.05). On the effects of N, P, and their interactions on soil properties, bacterial 16S rRNA gene abundance, and alpha diversity indices were examined by two-way ANOVA. Spearman’s correlation coefficient was used to test the relationships among soil properties, bacterial 16S rRNA gene abundance, alpha diversity indices, and abundant phyla. A *p* < 0.05 was considered to indicate significance.

Principle coordinates analysis (PCoA) was conducted to assess the dissimilarities of the bacterial community composition between the treatments using R 3.4 software (R Core Team, 2013). Per-mutational multivariate ANOVA (PERMANOVA) was further conducted to estimate the effects of N with P addition and their interactions on the composition of bacterial communities. A Mantel test was conducted to assess the correlation between specific soil properties and bacterial community composition. The canonical correspondence analysis (CCA) was performed to explore the effects of soil properties on bacterial community composition.

## Results

### Effects of N and/or P Addition on Soil Physicochemical Properties

The 6-year fertilization management significantly altered soil properties such as pH, NH_4_^+^-N, NO_3_^−^-N, TN, and AP contents. These results indicated that pH remarkably decreased in response to N amendment, while was not significantly altered after P amendment (**Table 1**). The concentrations of NH_4_^+^-N, NO_3_^−^-N, and TN were significantly lower in both the CK and P treatments than the N addition treatments. However, the increases in NH_4_^+^-N, NO_3_^−^-N, and TN concentrations did not significantly differ between N and NP treatments (*p* > 0.05). Soil AP content was significantly elevated by P addition. Two-way ANOVA revealed no significant interactions between N and P additions on the soil properties (*p* > 0.05).

**Table 1:** Effects N and/or P addition on soil properties under CK (no fertilizer), P fertilization (P), N fertilization (N), and NP fertilization (NP) treatments.

Treatment	pH (H_2_O)	NH_4_^+^-N (mg kg^−1^)	NO_3_^−^-N (mg kg^−1^)	DOC (mg kg^−1^)	Total N (g kg^−1^)	Available P (mg kg^−1^)
CK	4.69 ± 0.07^*a*^	4.97 ± 0.20^*b*^	1.27 ± 0.33^*b*^	64.79 ± 1.26^*a*^	1.03 ± 0.04^*b*^	3.14 ± 0.20^*b*^
P	4.63 ± 0.03^*a*^	4.72 ± 0.33^*b*^	1.26 ± 0.13^*b*^	63.59 ± 1.57^*a*^	1.06 ± 0.04^*b*^	16.23 ± 0.83^*a*^
N	3.95 ± 0.05^*b*^	10.98 ± 0.66^*a*^	5.30 ± 0.29^*a*^	66.11 ± 2.27^*a*^	1.51 ± 0.09^*a*^	3.09 ± 0.51^*b*^
NP	4.02 ± 0.06^*b*^	10.72 ± 0.17^*a*^	5.26 ± 0.43^*a*^	66.13 ± 2.23^*a*^	1.53 ± 0.12^*a*^	16.35 ± 1.27^*a*^
Significance of						
N	**<0.001**	**<0.001**	**<0.001**	0.215	**<0.001**	0.853
P	0.115	0.258	0.391	0.834	0.596	**<0.001**
N × P	0.164	0.775	0.054	0.218	0.859	0.927

*Different letters indicate significant difference among fertilizer treatments at p < 0.05 using two-way ANOVA. Significant values are shown in bold (p < 0.05). DOC, Dissolved organic carbon.*

### Effects of N and/or P Addition on Bacterial 16S rRNA Gene Copy Numbers

**FIGURE 1:**
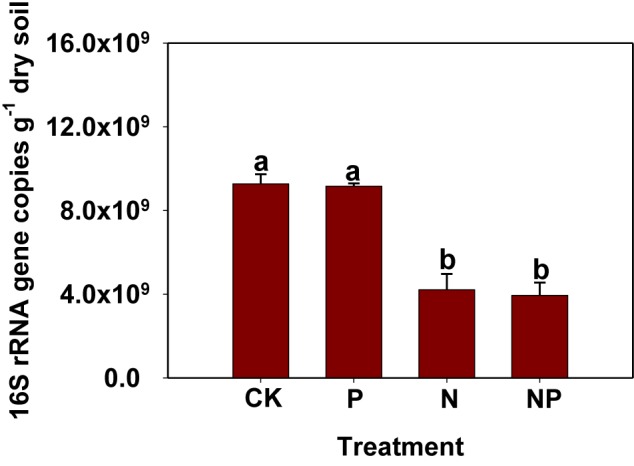
Abundance of bacteria as indicated by 16S rRNA gene copies examined by real-time PCR. Same letters above columns indicate no significant difference (*p* < 0.05). CK, no fertilizer; P, phosphorus input; N, nitrogen input; NP, nitrogen and phosphorus input.

The 6-year N and P inputs affected bacterial abundance as estimated based on qPCR of the bacterial 16S rRNA gene (**Figure 1**). Specifically, the abundance of bacterial 16S rRNA gene ranged from 3.95 × 10^9^ to 9.27 × 10^9^ copies g^−1^ dry soil. The bacterial abundance in the N and NP treatments was lower than in CK and P treatments, whereas no significant differences were observed in the CK and P treatments. 16S rRNA gene copies numbers were positively correlated with soil pH (*p* < 0.001), while negatively correlated with soil NH_4_^+^-N, NO_3_^−^-N, and TN (*p* < 0.001; Supplementary Table S1).

### Bacterial Alpha Diversity

Bacterial community analysis of the 16 soil samples revealed a total of 493,859 high quality sequences with 25,504 to 41,155 sequences per sample (Supplementary Table S2). To compare soil bacterial community diversity among all soils, the same survey effort level of 25,000 sequences were randomly selected from each sample in the sequencing library. The alpha diversity of the bacterial community was then examined using various estimators of richness and diversity (**Table 2**). There were significant differences in bacterial alpha diversity for the observed OTU numbers, as well as Chao 1, ACE, and Shannon’s index values among treatments, but not for Pielou’s Evenness among the treatments. Pielou’s evenness was high among treatments, indicating that bacterial community structure was evenly distributed. The observed OTU numbers, Chao 1, ACE, and Shannon’s index values were much lower in the N and NP treatments than in the CK treatment. Moreover, there were no significant (*p* > 0.05) differences in these alpha diversity indexes between CK and P addition treatments.

**Table 2:** Effects of N and/or P addition on soil bacterial alpha diversity indices.

Treatment	OTUs	Pielou’ Evenness	Richness		Diversity
			
			ACE	Chao1	Shannon
CK	1102 ± 53^*a*^	0.83 ± 0.02^*a*^	1535 ± 61^*a*^	1493 ± 109^*a*^	5.75 ± 0.10^*a*^
P	1123 ± 56^*a*^	0.82 ± 0.01^*a*^	1520 ± 35^*a*^	1530 ± 25^*a*^	5.77 ± 0.07^*a*^
N	902 ± 36^*b*^	0.81 ± 0.01^*a*^	1304 ± 74^*b*^	1170 ± 94^*b*^	5.32 ± 0.19^*b*^
NP	851 ± 104^*b*^	0.82 ± 0.02^*a*^	1332 ± 70^*b*^	1213 ± 58^*b*^	5.41 ± 0.09^*b*^

*OTUs, operational taxonomic units (97% similarity). Pielou’ Evenness: Pielou’ Evenness = H′/ln(S) where H′ is Shannon diversity and S is the total number of species in a sample. Based on Chao1 and abundance-based coverage estimator (ACE) richness indices. Based on Shannon diversity indices. Data are means ± standard deviation (n = 4). Different letters above columns indicate significant difference among fertilizer treatments (p < 0.05). CK, no fertilizer; P, phosphorus input; N, nitrogen input; NP, nitrogen and phosphorus input.*

The correlations of bacterial alpha diversity indices with soil physicochemical parameters are shown in **Table 3**. The values of observed OTU numbers, Chao1, ACE, and Shannon’s index were positively correlated with soil pH (*p* < 0.01), but were significantly and negatively correlated with NH_4_^+^-N, NO_3_^−^-N, and TN contents (*p* < 0.05).

**Table 3:** Spearman’s correlations between soil properties and alpha diversity.

	pH	NH_4_^+^-N	NO_3_^−^-N	DOC	TN	AP
OTU numbers	0.740^∗∗^	−0.703^∗∗^	−0.675^∗∗^	−0.438	−0.693^∗∗^	0.152
Chao1	0.898^∗∗∗^	−0.871^∗∗∗^	−0.848^∗∗∗^	−0.449	−0.831^∗∗∗^	0.115
ACE	0.852^∗∗∗^	−0.831^∗∗∗^	−0.791^∗∗∗^	−0.513	−0.818^∗∗∗^	0.086
Shannon index	0.687^∗∗^	−0.682^∗∗∗^	−0.601^∗^	−0.332	−0.605^∗∗^	0.121

*^∗∗∗^p < 0.001, ^∗∗^p < 0.01, ^∗^p < 0.05. DOC, Dissolved organic carbon.*

### Changes in Bacterial Community Composition in Response to N and/or P Addition

All reads in each treatment were classified into eight phyla, and their relative abundances were shown in **Figure 4A**. The phyla *Acidobacteria* and *Proteobacteria* were most abundant in all treatments, comprising 67.1–74.3% of the bacterial sequences obtained from the soil, followed by *Chloroflexi* (8.9–9.8%), *Actinobacteria* (4.9–10.5%), *Verrucomicrobia* (2.1–2.9%), *Planctomycetes* (1.3–2.2%), and *Bacteroidetes* (1.7–2.1%). Different fertilization management strategies had various influences on bacterial composition at the phylum level. The relative abundance of the phyla *Actinobacteria* and *Proteobacteria* increased in N and NP treatments, while that of *Acidobacteria* decreased, relative to the CK and P treatments. There were no significant differences in the abundance of *Acidobacteria*, *Actinobacteria*, *Proteobacteria*, *Chloroflexi*, *Actinobacteria*, *Planctomycetes*, *Bacteroidetes*, and *Verrucomicrobia* between N and NP treatments. Similarly, the relative abundance of these phyla did not differ significantly between CK and P treatments (*p* > 0.05).

At class level, the 13 most abundant classes were obtained (>2%) among fertilizer treatments (**Figure 4B**). There were significant differences between treatments for the four most abundant classes. Specifically, the relative abundance of class *Acidobacteria* was higher in the CK and P treatments than that in the N-related treatments. Furthermore, the relative abundance of *Actinobacteria*, *Alphaproteobacteria*, and *Gammaproteobacteria* significantly increased in the N and NP treatments.

**FIGURE 2:**
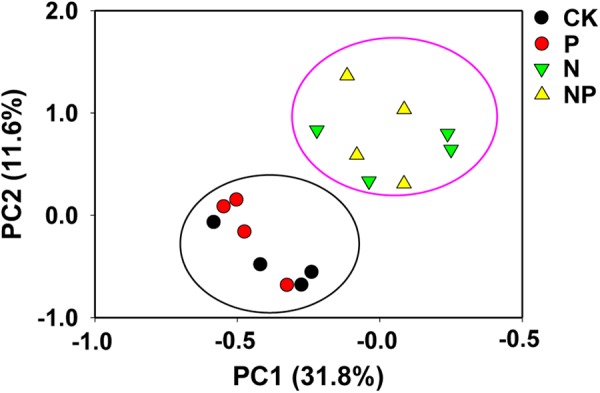
Principle coordinate analysis (PCoA) to visualize the overall differences in bacterial community composition across the all treatments. CK, no fertilizer; P, phosphorus input; N, nitrogen input; NP, nitrogen and phosphorus input.

The variations in bacterial communities caused by N and/or P input were evaluated by PCoA (**Figure 2**), which revealed that bacterial community composition separated clearly between N and CK treatments, while the bacterial community composition in the P treatment was similar to that in the CK treatment. The first two principal coordinates explained 31.8% (PC1) and 11.6% (PC2) of the variation in bacterial communities, respectively. PERMANOVA further revealed significant differences between N-free treatments (CK and P) and N-containing treatments (N and NP) (*p* < 0.01; Supplementary Table S2).

### The Relationship Between Bacterial Community Structure and Soil Properties

**FIGURE 3:**
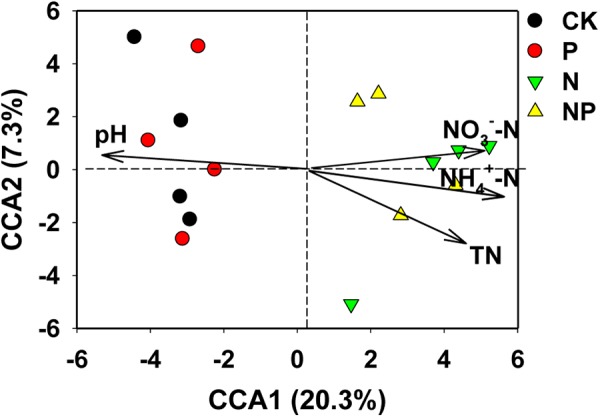
Canonical correspondence analysis (CCA) illustrating the effects of soil properties on bacterial community structure. CK, no fertilizer; P, phosphorus input; N, nitrogen input; NP, nitrogen and phosphorus input.

The Mantel test indicated that bacterial community structure was closely correlated with soil chemical properties including pH, NH_4_^+^-N, NO_3_^−^-N, and TN (*p* < 0.01), and the correlation coefficients followed the trend: pH > NH_4_^+^-N > NO_3_^−^-N > TN (Supplementary Table S4). The effects of these soil properties on the bacterial community structure were further analyzed using CCA (**Figure 3**), which revealed that these soil variables explained 27.6% of the variation in the bacterial community by the first two constrained axes of CCA, with the first axis explaining 20.3% and the second 7.3%. Moreover, CCA clearly showed that soil pH and mineral N contents were the most important contributors to the variation in bacterial communities, and the direction of these arrows was closely correlated with the X axis.

We also evaluated the correlations between relative abundant phyla (>2%) and soil properties (**Table 4**). Three dominant phyla (*Acidobacteria*, *Proteobacteria*, and *Actinobacteria*) were significantly correlated with several soil chemical parameters (*p* < 0.05), while *Bacteroidetes*, *Verrucomicrobia*, *Chloroflexi*, and *Planctomycetes* were not obviously related to any of the soil chemical properties (*p* > 0.05). Only *Acidobacteria* was positively correlated with soil pH, whereas *Actinobacteria* and *Proteobacteria* were negatively correlated with this factor (*p* < 0.05). Moreover, *Acidobacteria* was negatively related to NH_4_^+^-N, NO_3_^−^-N, and TN, while *Actinobacteria* and *Proteobacteria* were positively correlated with these soil properties.

**Table 4:** Spearman’s correlation coefficients between soil chemical characteristics and abundant phyla (relative abundance > 2%).

	pH	NH_4_^+^-N	NO_3_^−^-N	DOC	TN	AP
*Acidobacteria*	0.898^∗∗∗^	−0.875^∗∗∗^	−0.829^∗∗∗^	−0.426	−0.814^∗∗∗^	0.13
*Actinobacteria*	−0.844^∗∗∗^	0.838^∗∗∗^	0.807^∗∗∗^	0.498*	0.803^∗∗∗^	−0.061
*Bacteroidetes*	0.064	−0.14	−0.116	0.109	0.208	0.091
*Chloroflexi*	−0.241	0.309	0.381	−0.02	0.433	0.336
*Planctomycetes*	0.507	−0.352	−0.562	−0.492	−0.516	−0.474
*Proteobacteria*	−0.884^∗∗∗^	0.817^∗∗∗^	0.735**	0.395	0.749^∗∗∗^	0.016
*Verrucomicrobia*	0.054	0.037	−0.017	0.163	0.141	0.237
*WD272*	−0.287	0.237	0.064	0.077	0.233	−0.057

*^∗∗∗^p < 0.001, ^∗∗^p < 0.01, ^∗^p < 0.05. DOC, Dissolved organic carbon.*

## Discussion

### Influence of N and/or P Amendment on Bacterial 16S rRNA Gene Abundance and α-Diversity

The applications of inorganic N were previously believed to reduce microbial abundance and biodiversity (He et al., 2007; Geisseler and Scow, 2014; Zhou et al., 2015; Ling et al., 2017; Wang et al., 2017; Nie et al., 2018). Similarly, N amendment (such as N and NP treatments) significantly decreased bacterial 16S rRNA gene abundance and biodiversity (**Figure 1** and **Table 2**). Conversely, long-term P input did not affect bacterial abundance and α-diversity, which was supported by the result of recent studies (Huang et al., 2016; Liu et al., 2018). Huang et al. (2016) reported that the abundance of most groups of soil microbial community (bacteria, fungal, and AMF) was not affected by the low (5 g P m^−2^ year^−1^) and medium (5 g P m^−2^ year^−1^) P inputs. Additionally, Liu et al. (2018) found that bacterial Chao1 and Shannon indices were not strongly influenced by P amendment. Thus, our results conclude that P availability may not be the limiting factor affecting bacterial abundance and biodiversity in this Chinese fir plantation. Changes of bacterial 16S rRNA gene abundance and α-diversity might be mainly associated with the soil pH, as soil pH was kept at a relative stable level in the P treatment, while it decreased remarkably under N-related treatments. Particularly, the spearman’s correlation analysis showed that bacterial 16S rRNA gene abundance, and α-diversity indices were strongly correlated with soil pH in the present study, indicating that soil pH was a decisive factor affecting them. Moreover, the bacterial community was significantly influenced by soil pH because most bacterial taxa showed relatively narrow growth tolerances, especially within the pH range of 4–7 (Rousk et al., 2010; Dai et al., 2018). Additionally, bacterial 16S rRNA gene abundance and α-diversity were closely related to NH_4_^+^-N and NO_3_^−^-N rather than AP, further confirming that mineral N availability was a very important factor altering bacterial abundance and biodiversity (Zhou et al., 2015; Nie et al., 2018; Wang et al., 2018). Therefore, we concluded that the change of bacterial abundance and biodiversity among the all treatments could be attributed to the direct influence of N fertilizer as nutrients and indirect effects of soil acidification caused by N input.

### Responses of Dominant Bacterial Community to N and/or P Inputs

In the present study, high-throughput sequencing analysis indicated that *Proteobacteria*, *Acidobacteria*, *Chloroflexi*, and *Actinobacteria* were the predominant bacterial phyla in the acidic forest soil, which was in agreement with the results of previous studies (Sun et al., 2015; Cui et al., 2017; Nie et al., 2018). Our results revealed that N input increased the relative abundance of *Proteobacteria* and *Actinobacteria* and reduced the relative abundance of *Acidobacteria*. These findings were supported by the copiotrophic hypothesis (Fierer et al., 2007), in which copiotrophic groups (e.g., *Proteobacteria* and *Actinobacteria*) that had fast growth rates were more likely to increase in nutrient-rich conditions, while oligotrophic groups (e.g., *Acidobacteria* and *Chloroflexi*) that had slower growth rates would likely decline (Fierer et al., 2012). *Proteobacteria* and *Actinobacteria* have been reported to be related to the high carbon availability, and these organisms exhibit relatively rapid growth rates. Meanwhile, *Acidobacteria* belongs to the group of oligotrophic bacteria found in nutrient poor and very acidic environments and have the ability to degrade complex and recalcitrant carbon compounds (Ai et al., 2015). However, P input did not follow this rule to affect the relative abundances of selected oligotrophic and copiotrophic taxa, which was consistent with recent study reported by Wakelin et al. (2017). We concluded soil available phosphorus content was not sufficient to affect bacterial community in our study. When soil available phosphorus content was ≥100 mg kg^−1^, soil-borne copiotrophic bacteria members became dominant (Kuramae et al., 2011). However, soil available phosphorus content was not reached in our study and the red soil was different from that by Kuramae et al. (2011). Additionally, the KEGG Orthology (K) numbers showed a significant difference among the fertilizer treatments using functional predictions analysis (Supplementary Figure S1). 16S rRNA gene-predicted functional structure was more affected by N addition than P input. Some functions related to amino acid metabolism, carbohydrate metabolism, energy metabolism, and xenobiotics biodegradation and metabolism were reduced in samples receiving inorganic N addition. Thus, the different response of major phyla to N and P input might be reason for the change in bacterial community structure.

### Effects of N and/or P Inputs on the Bacterial Community Structure

**FIGURE 4:**
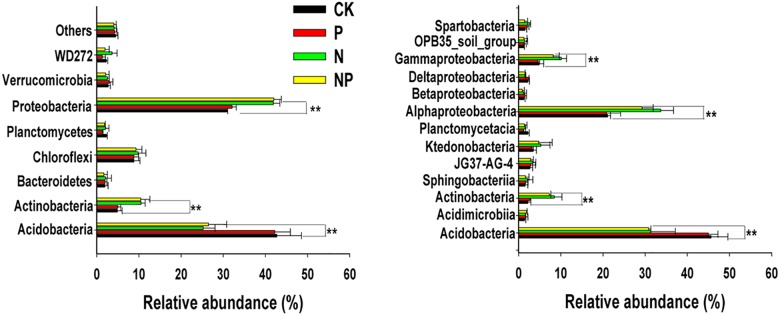
Relative abundances of the most abundance bacterial groups in different treatments at the phyla **(A)** and class **(B)** levels. Others represented unclassified groups. Data are means ± standard deviation (*n* = 4). Significance is indicated by ^∗∗^*p* < 0.01. CK, no fertilizer; P, phosphorus input; N, nitrogen input; NP, nitrogen and phosphorus input.

Long-term inorganic fertilization, especially N fertilizer, usually changes soil microbial community structure in previous studies (Zhou et al., 2015, 2017; Nie et al., 2018). Similarly, N input altered bacterial community structure in our study (**Figure 4**). Bacterial community composition in the N and NP treatments differed significantly from those not receiving N (CK and P), indicating that fertilizer addition had a stronger influence on it than P input. On the contrary, P input did not influence bacterial community structure in the present study, which was supported by previous reports (Huang et al., 2016; Liu et al., 2018). Huang et al. (2016) and Liu et al. (2018) observed no changes in soil bacterial community composition among the various P treatments by phospholipid fatty acids (PFLA) and high throughput sequencing analyses, respectively. In addition, Ling et al. (2017) and Leff et al. (2015) found that high N availability, affected by N input, was a key factor influencing shifts in bacterial community structure. Nitrogen fertilizer directly impacted mineral N availability, which were the key factors altering bacterial community composition (**Figure 3**), as revealed in previous studies (Zhong et al., 2015; Nie et al., 2018). Taken together, these findings indicate that soil mineral N availability, which depends on N input, is essential to the bacterial community structure.

In addition to soil N availability, pH is frequently considered to be another important factor in controlling bacterial community structure and is considered a good predictor of bacterial community composition (Geisseler and Scow, 2014; Li et al., 2014), which was also observed in the present sturdy. Nitrogen rather than P inputs led to decrease in soil pH, and soil pH was more important than other nutrients in shaping bacterial community structure according to the Mantel test and CCA analysis, which is supported by previous studies (Zhou et al., 2015; Xun et al., 2016; Wang et al., 2018). Moreover, the most abundant phyla were more tightly related to pH than other nutrients (Supplementary Table S3). These results further supported that the changes in soil pH caused by the application of N fertilizer were more important than available nutrients on bacterial community structure.

## Conclusion

Soil chemical properties and bacterial community were markedly influenced by N and/or P input in a subtropical fir plantation in southern China. Our results showed that N input influenced bacterial abundance and community composition while P input did not. The shift in soil pH induced by of the application of N alone and N plus P fertilizer must be a decisive factor in determining bacterial abundance and community structure. In addition to soil pH, soil mineral N availability also appeared to change bacterial community structure. Our findings indicated that N input reduced bacterial abundance and diversity, and that the impacts of N amendment (N and NP) treatments were stronger than treatments that did not contain (P). This study provides valuable information that improves our understanding of the effects of N and/or P input on underground bacterial community and revealed the main factors influencing bacterial communities in subtropical forest ecosystems.

## Author Contributions

QW and CW contributed to the design and implementation of the research, participated in drafting the manuscript. WY contributed to analyze the results. AT, DC, YH, JA, YJ, and ZH revised the manuscript.

## Conflict of Interest Statement

The authors declare that the research was conducted in the absence of any commercial or financial relationships that could be construed as a potential conflict of interest.

## References

[B1] Ai C. Liang G. Sun J. Wang X. He P. Zhou W. He X. (2015). Reduced dependence of rhizosphere microbiome on plant-derived carbon in 32-year long-term inorganic and organic fertilized soils. *Soil Biol. Biochem.* 80 70–78. 10.1016/j.soilbio.2014.09.028

[B2] Artursson V. Finlay R. D. Jansson J. K. (2006). Interactions between arbuscular mycorrhizal fungi and bacteria and their potential for stimulating plant growth. *Environ. Microbiol.* 8 1–10. 10.1111/j.1462-2920.2005.00942.x 16343316

[B3] Bending G. D. Turner M. K. Rayns F. Marx M. C. Wood M. (2004). Microbial and biochemical soil quality indicators and their potential for differentiating areas under contrasting agricultural management regimes. *Soil Biol. Biochem.* 36 1785–1792. 10.1016/j.soilbio.2004.04.035

[B4] Bobbink R. Hicks K. Galloway J. Spranger T. Alkemade R. Ashmore M. (2010). Global assessment of nitrogen deposition effects on terrestrial plant diversity: a synthesis. *Ecol. Appl.* 20 30–59. 10.1890/08-1140.1 20349829

[B5] Chen F. S. Niklas K. J. Liu Y. Fang X. M. Wan S. Z. Wang H. M. (2015). Nitrogen and phosphorus additions alter nutrient dynamics but not resorption efficiencies of Chinese fir leaves and twigs differing in age. *Tree Physiol.* 35 1106–1117. 10.1093/treephys/tpv076 26358049

[B6] Cleveland C. C. Reed S. C. Townsend A. R. (2006). Nutrient regulation of organic matter decomposition in a tropical rain forest. *Ecology* 87 492–503. 10.1890/05-0525 16637373

[B7] Coleman D. C. Whitman W. B. (2005). Linking species richness, biodiversity and ecosystem function in soil systems. *Pedobiologia* 49 479–497. 10.1016/j.pedobi.2005.05.006

[B8] Cui J. Wang J. J. Xu J. Xu C. H. Xu X. N. (2017). Changes in soil bacterial communities in an evergreen broad-leaved forest in east China following 4 years of nitrogen addition. *J. Soils Sediments* 17:2156 10.1007/s11368-017-1671-y

[B9] Dai Z. M. Su W. Q. Chen H. H. Barberán A. Zhao H. C. Yu M. J. (2018). Long-term nitrogen fertilization decreases bacterial diversity and favors the growth of *Actinobacteria* and *Proteobacteria* in agro-ecosystems across the globe. 10.1111/gcb.14163 29645398

[B10] Dentener F. Drevet J. Lamarque J. F. Bey I. Eickhout B. Fiore A. M. (2006). Nitrogen and sulfur deposition on regional and global scales: a multimodel evaluation. *Glob. Biogeochem. Cycles* 20 16615 10.1029/2005GB002672

[B11] Dong W. Y. Zhang X. Y. Liu X. Y. Fu X. L. Chen F. S. Wang H. M. (2015). Responses of soil microbial communities and enzyme activities to nitrogen and phosphorus additions in Chinese fir plantations of subtropical China. *Biogeosciences* 12 5537–5546. 10.5194/bg-12-5537-2015

[B12] Dunbar J. Barns S. M. Ticknor L. O. Kuske C. R. (2002). Empirical and theoretical bacterial diversity in four Arizona soils. *Appl. Environ. Microbiol.* 68 3035–3045. 10.1128/AEM.68.6.3035-3045.2002 12039765 PMC123964

[B13] Edgar R. C. (2013). UPARSE: highly accurate OTU sequences from microbial amplicon reads. *Nat. Methods* 10 996–998. 10.1038/nmeth.2604 23955772

[B14] Fan H. B. Wu J. P. Liu W. F. Yuan Y. H. Huang R. Z. Liao Y. C. (2014). Nitrogen deposition promotes ecosystem carbon accumulation by reducing soil carbon emission in a subtropical forest. *Plant Soil* 379 361–371. 10.1007/s11104-014-2076-y

[B15] Fierer N. Jackson J. A. Vilgalys R. Jackson R. B. (2005). Assessment of soil microbial community structure by use of taxon-specific quantitative pcr assays. *Appl. Environ. Microbiol.* 71 4117–4120. 10.1128/AEM.71.7.4117-4120.2005 16000830 PMC1169028

[B16] Fierer N. Bradford M. A. Jackson R. B. (2007). Toward an ecological classification of soil bacteria. *Ecology* 88 1354–1364. 10.1890/05-1839 17601128

[B17] Fierer N. Lauber C. L. Ramirez K. S. Zaneveld J. Bradford M. A. Knight R. (2012). Comparative metagenomic, phylogenetic and physiological analyses of soil microbial communities across nitrogen gradients. *ISME J.* 6 1007–1017. 10.1038/ismej.2011.159 22134642 PMC3329107

[B18] Freedman Z. B. Romanowicz K. J. Upchurch R. A. Zak D. R. (2015). Differential responses of total and active soil microbial communities to long-term experimental N deposition. *Soil Biol. Biochem.* 90 275–282. 10.1016/j.soilbio.2015.08.014

[B19] Geisseler D. Scow K. M. (2014). Long-term effects of mineral fertilizers on soil microorganisms-A review. *Soil Biol. Biochem.* 75 54–63. 10.1016/j.soilbio.2014.03.023

[B20] Guo J. H. Liu X. J. Zhang Y. Shen J. L. Han W. X. Zhang W. F. (2010). Significant acidification in major Chinese croplands. *Science* 327 1008–1010. 10.1126/science.1182570 20150447

[B21] He J. Z. Shen J. P. Zhang L. M. Zhu Y. G. Zheng Y. M. Xu M. G. (2007). Quantitative analyses of the abundance and composition of ammonia-oxidizing bacteria and ammonia-oxidizing archaea of a Chinese upland red soil under long-term fertilization practices. *Environ. Microbiol.* 9 2364–2374. 10.1111/j.1462-2920.2007.01358.x 17686032

[B22] Huang J. Hu B. Qi K. Chen W. Pang X. Bao W. (2016). Effects of phosphorus addition on soil microbial biomass and community composition in a subalpine spruce plantation. *Eur. J. Soil Biol.* 72 35–41. 10.1016/j.ejsobi.2015.12.007

[B23] IPCC (2007). “Climate change 2007: the physical science basis,” in *Contribution of Working Group I to the Fourth Assessment Report of the Intergovernmental Panel on Climate Chang*, eds Solomon S. Qin D. Manning M. (Cambridge: Cambridge University Press).

[B24] IPCC (2013). “Climate change 2013: the physical science basis,” in *Contribution of Working Group I to the Fifth Assessment Report of the Intergovernmental Panel on Climate Change*, eds Stocker T. F. Qin D. Plattner G. K. Tignor M. Allen S. K. Boschung J. (Cambridge: Cambridge University Press).

[B25] Kaspari M. Garcia M. N. Harms K. E. Santana M. Wright S. J. Yavitt J. B. (2008). Multiple nutrients limit litter-fall and decomposition in a tropical forest. *Ecol. Lett.* 11 35–43. 10.1111/j.1461-0248.2007.01124.x 18021246

[B26] Kuramae E. Gamper H. Jv V. Kowalchuk G. (2011). Soil and plant factors driving the community of soil-borne microorganisms across chronosequences of secondary succession of chalk grasslands with a neutral pH. *FEMS Microbiol. Ecol.* 77 285–294. 10.1111/j.1574-6941.2011.01110.x 21488909

[B27] Leff J. W. Jones S. E. Prober S. M. Barberán A. Borer E. T. Firn J. L. (2015). Consistent responses of soil microbial communities to elevated nutrient inputs in grasslands across the globe. *Proc. Natl. Acad. Sci. U.S.A.* 112:10967. 10.1073/pnas.1508382112 26283343 PMC4568213

[B28] Li C. Yan K. Tang L. Jia Z. Li Y. (2014). Change in deep soil microbial communities due to long-term fertilization. *Soil Biol. Biochem.* 75 264–272. 10.1016/j.soilbio.2014.04.023

[B29] Ling N. Chen D. Guo H. Wei J. X. Bai Y. F. Shen Q. R. (2017). Differential responses of soil bacterial communities to long-term N and P inputs in a semi-arid steppe. *Geoderma* 292 25–33. 10.1016/j.geoderma.2017.01.013

[B30] Liu M. Liu J. Chen X. Jiang C. Wu M. Li Z. (2018). Shifts in bacterial and fungal diversity in a paddy soil faced with phosphorus surplus. *Biol. Fertil. Soils* 54 259–267. 10.1007/s00374-017-1258-1

[B31] Lu X. K. Mo J. M. Gilliam F. S. Zhou G. Y. Fang Y. T. (2010). Effects of experimental nitrogen additions on plant diversity in an old-growth tropical forest. *Glob. Change Biol.* 16 2688–2700. 10.1111/j.1365-2486.2010.02174.x

[B32] Mao Q. G. Lu X. K. Zhou K. J. Chen H. Zhu X. M. Mori T. K. (2017). Effects of long-term nitrogen and phosphorus additions on soil acidification in an N-rich tropical forest. *Geoderma* 285 57–63. 10.1016/j.geoderma.2016.09.017

[B33] Mele P. M. Crowley D. E. (2008). Application of self-organizing maps for assessing soil biological quality. *Agric. Ecosyst. Environ.* 126 139–152. 10.1016/j.agee.2007.12.008

[B34] Morris S. J. Blackwood C. B. (2015). “The ecology of the soil biota and their function,” *in Soil Microbiology, Ecology and Biochemistry*, 4th Edn, Chap. 10 ed. Eldor E. A. (Cambridge, MA: Academic Press), 273–309. 10.1016/B978-0-12-415955-6.00010-4

[B35] Nie Y. Wang M. Zhang W. Ni Z. Hashidoko Y. Shen W. (2018). Ammonium nitrogen content is a dominant predictor of bacterial community composition in an acidic forest soil with exogenous nitrogen enrichment. *Sci. Total Environ.* 624 407–415. 10.1016/j.scitotenv.2017.12.142 29262382

[B36] R Core Team (2013). *R: A Language and Environment for Statistical Computing.R Foundation for Statistical Computing, Vienna, Austria*. Available at: http://www.R-project.org/

[B37] Rousk J. Bååth E. Brookes P. C. Lauber C. L. Lozupone C. Caporaso J. G. (2010). Soil bacterial and fungal communities across a pH gradient in an arable soil. *ISME J.* 4:1340. 10.1038/ismej.2010.58 20445636

[B38] Schloss P. D. Westcott S. L. Ryabin T. Hall J. R. Hartmann M. Hollister E. B. (2009). Introducing mothur: open-source, platform-independent, community-supported software for describing and comparing microbial communities. *Appl. Environ. Microbiol.* 75 7537–7541. 10.1128/AEM.01541-09 19801464 PMC2786419

[B39] Sun R. Zhang X. X. Guo X. Wang D. Chu H. (2015). Bacterial diversity in soils subjected to long-term chemical fertilization can be more stably maintained with the addition of livestock manure than wheat straw. *Soil Biol. Biochem.* 88 9–18. 10.1016/j.soilbio.2015.05.007

[B40] Tang Y. Q. Zhang X. Y. Li D. D. Wang H. M. Chen F. S. Fu X. L. (2016). Impacts of nitrogen and phosphorus additions on the abundance and community structure of ammonia oxidizers and denitrifying bacteria in Chinese fir plantations. *Soil Biol. Biochem.* 103 284–293. 10.1016/j.soilbio.2016.09.001

[B41] van der Heijden M. G. Bardgett R. D. Van Straalen N. M. (2008). The unseen majority: soil microbes as drivers of plant diversity and productivity in terrestrial ecosystems. *Ecol. Lett.* 11 296–310. 10.1111/j.1461-0248.2007.01139.x 18047587

[B42] Wang C. Zheng M. Song W. Wen S. Wang B. Zhu C. (2017). Impact of 25 years of inorganic fertilization on diazotrophic abundance and community structure in an acidic soil in southern China. *Soil Biol. Biochem.* 113 240–249. 10.1016/j.soilbio.2017.06.019

[B43] Wang Q. Garrity G. M. Tiedje J. M. Cole J. R. (2007). Naive Bayesian classifier for rapid assignment of rRNA sequences into the new bacterial taxonomy. *Appl. Environ. Microbiol.* 73 5261–5267. 10.1128/AEM.00062-07 17586664 PMC1950982

[B44] Wang Q. F Jiang X. Guan D. Wei D. Zhao B. Chen S. (2018). Long-term fertilization changes bacterial diversity and bacterial communities in the maize rhizosphere of Chinese Mollisols. *Appl. Soil Ecol.* 125 88–96. 10.1016/j.apsoil.2017.12.007

[B45] Watkins K. L. Veum T. L. Krause G. F. (1987). Total nitrogen determination of various dample types: a comparison of the Hach, Kjeltec, and Kjeldahl methods. *J. Assoc. Off. Anal. Chem.* 70 410–412. 3610952

[B46] Wakelin S. A. Condron L. M. Gerard E. Dignam B. E. A. Black A. O’Callaghan M. (2017). Long-term P fertilisation of pasture soil did not increase soil organic matter stocks but increased microbial biomass and activity. *Biol. Fert. Soils* 53 511–521. 10.1007/s00374-017-1212-2 11706800

[B47] Weand M. P. Arthur M. A. Lovett G. M. McCulley R. L. Weathers K. C. (2010). Effects of tree species and N additions on forest floor microbial communities and extracellular enzyme activities. *Soil Biol. Biochem.* 42 2161–2173. 10.1016/j.soilbio.2010.08.012

[B48] Wei X. Blanco J. A. Jiang H. Kimmins J. H. (2012). Effects of nitrogen deposition on carbon sequestration in Chinese fir forest ecosystems. *Sci. Total Environ.* 416 351–361. 10.1016/j.scitotenv.2011.11.087 22225819

[B49] Xun W. Zhao J. Xue C. Zhang G. Ran W. Wang B. (2016). Significant alteration of soil bacterial communities and organic carbon decomposition by different long-term fertilization management conditions of extremely low-productivity arable soil in South China. *Environ. Microbiol.* 18 1907–1917. 10.1111/1462-2920.13098 26486414

[B50] Zhang X. Q. Kirschbaum M. U. Hou Z. Guo Z. (2004). Carbon stock changes in successive rotations of Chinese fir (*Cunninghamia lanceolata* (lamb) hook) plantations. *For. Ecol. Manag.* 202 131–147. 10.1016/j.foreco.2004.07.032

[B51] Zhao J. Wang F. Li J. Zou B. Wang X. Li Z. (2014). Effects of experimental nitrogen and/or phosphorus additions on soil nematode communities in a secondary tropical forest. *Soil Biol. Biochem.* 75 1–10. 10.1016/j.soilbio.2014.03.019

[B52] Zhong Y. Q. W. Yan W. M. ShangGuan Z. P. (2015). Impact of long-term N additions upon coupling between soil microbial community structure and activity, and nutrient-use efficiencies. *Soil Biol. Biochem.* 91 151–159. 10.1016/j.soilbio.2015.08.030

[B53] Zhong W. H. Gu T. Wang W. Zhang B. Lin X. G. Huang Q. R. (2010). The effects of mineral fertilizer and organic manure on soil microbial community and diversity. *Plant Soil* 326 511–522. 10.1007/s11104-009-9988-y

[B54] Zhou J. Guan D. W. Zhou B. K. Zhao B. S. Ma M. C. Qin J. (2015). Influence of 34-years of fertilization on bacterial communities in an intensively cultivated black soil in northeast China. *Soil Biol. Biochem.* 90 42–51. 10.1016/j.soilbio.2015.07.005

[B55] Zhou J. Jiang X. Wei D. Zhao B. Ma M. Chen S. (2017). Consistent effects of nitrogen fertilization on soil bacterial communities in black soils for two crop seasons in China. *Sci. Rep.* 7:3267. 10.1038/s41598-017-03539-6 28607352 PMC5468298

