# Supplementary material for: Effects of Nitrogen and Phosphorus Inputs on Soil Bacterial Abundance, Diversity, and Community Composition in Chinese Fir Plantations
Source: Front Microbiol. 2018 Jul 19;9:1543. doi: 10.3389/fmicb.2018.01543 (PMC6060263; doi:10.3389/fmicb.2018.01543)

Effects of nitrogen and phosphorus inputs on soil bacterial abundance, diversity and community composition in Chinese fir plantations

Qing Wang[a,b*], Cong Wang[c], WeiWei Yu[b], Ali Turak[d,*], Diwen Chen[a], Ying Huang[a], Junhua Ao[a,*], Yong Jiang[a], Zhenrui Huang[a]

[a] *Guangdong Provincial Bioengineering Institute (Guangzhou Sugarcane Industry Research Institute), Guangdong Key Laboratory of Sugarcane Improvement and Biorefinery, Guangzhou 510316, China.*

[b] *State Key Laboratory of Urban and Regional Ecology, Research Center for Eco-Environmental Sciences, Chinese Academy of Sciences, Beijing 100085, China.*

[c] *Guangdong Key Laboratory of Integrated Agro-environmental Pollution Control and Management, Guangdong Institute of Eco-environmental Science and Technology, Guangzhou 510650, PR China.*

[d] *Science and Technology Management Dept. Sinofert Holdings Ltd. Level 10, Central Tower, Chemsunny World Trade Center,28 FuxingmengNei Street, Beijing, P.R. 100031,China*

*Corresponding author: Qing Wang Email address：wangqing316000@163.com

*Corresponding author: Junhua Ao Email address：huangqiong870730@163.com

Table S1 Pearson correlation coefficients between soil properties and bacterial 16S rRNA gene abundance. Values in bold indicate significant correlations ($p < 0.05$).

	**r**	***p***
**pH**	0.915	**<0.001**
**NH_4^+-N**	-0.838	**<0.001**
**NO_3^--N**	-0.824	**<0.001**
**DOC**	-0.33	0.209
**TN**	-0.821	**<0.001**
**AP**	-0.034	0.986

Table S2 Raw sequence reads, trimmed reads and the average length of sequences (bp) across all soil samples.

Treatment	Plot number	Raw reads in total	Trimmed reads	Average length of sequences(bp)
CK	1	25778	25504	409.6
	2	28511	28071	412.1
	3	36557	35991	409.7
	4	33219	32857	406.8
P	5	42533	41159	408.1
	6	31403	30577	407.7
	7	36588	35797	401.9
	8	27957	27508	409.3
N	9	27214	26991	407.4
	10	27059	26707	406.1
	11	33976	32996	405.6
	12	31742	30529	410.5
NP	13	33976	32996	405.6
	14	28618	28016	410.6
	15	27612	27001	409.3
	16	39821	31159	409.4

Table S3 Results of PERMANOVA for testing the effects of N, P addition and their interactions on bacterial community structure. *p* values (p<0.05) are indicated in bold.

	**F**	***p***
**CK vs P**	0.737	0.868
**CK vs N**	2.597	**0.024**
**CK vs N+P**	2.577	**0.026**
**P vs N**	3.076	**0.026**
**P vs N+P**	2.696	**0.023**
**N vs N+P**	0.884	0.413

Table S4 Relationships of soil bacterial community composition with soil chemical variables, as revealed by Mantel test. Values in bold indicate significant correlations (p< 0.05).

Variable	**pH**	**NH_4^+-N**	**NO_3^--N**	**DOC**	**TN**	**AP**
**r**	0.46	0.38	0.33	0.25	0.32	-0.055
p	**0.003**	**0.002**	**0.004**	0.054	**0.003**	0.69

FgureS1 Effects of N and/or P addition on the KEGG Orthology numbers, which was used for functional predictions. Data are means ± standard deviation (n=4). Significance is indicated by $**p<0.01$

Figure S1

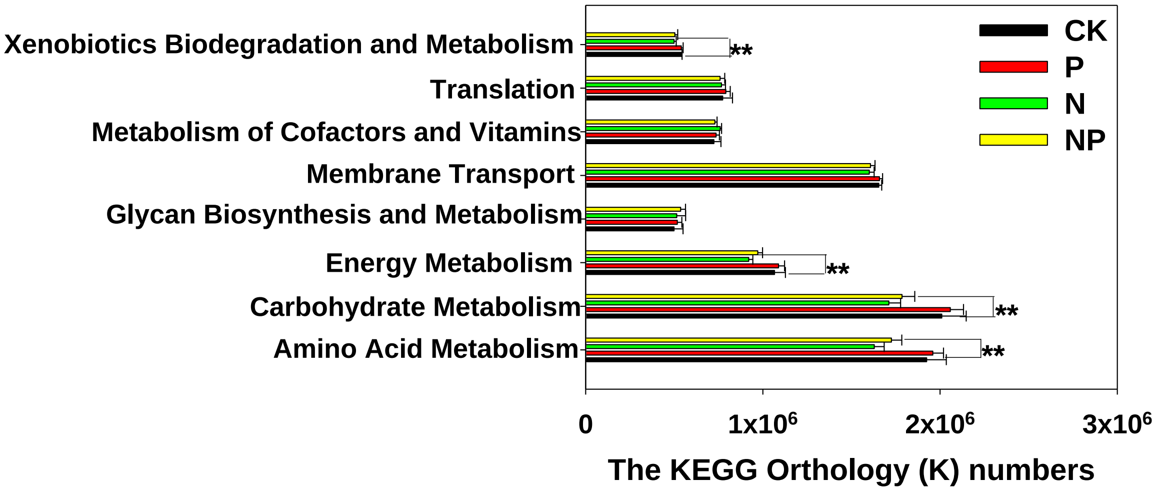

Supplement: Supplementary file 1 [file Data_Sheet_1.pdf]
